# A Remarkable New Species of the Genus *Paraglenea* Bates from China, with Notes on the Genus *Malloderma* Lacordaire (Coleoptera: Cerambycidae: Lamiinae: Saperdini)

**DOI:** 10.3390/insects16080867

**Published:** 2025-08-21

**Authors:** Mei-Ying Lin, Ren-Jie You, Ling-Yun Wang

**Affiliations:** 1 Engineering Research Center for Forest and Grassland Disaster Prevention and Reduction/Ecological Security and Protection Key Laboratory of Sichuan Province, School of Life Sciences (School of Ecological Forestry), Mianyang Normal University, 166 Mianxing West Road, Mianyang 621000, China; 2 Independent Researcher, 8 Jiefangjie, Lufengzhen, Xupu, Huaihua 419300, China

**Keywords:** new species, longhorn beetle, new distribution record, male genitalia, male claws, taxonomy

## Abstract

This study contributes to the taxonomic research on the tribe Saperdini, focusing on two genera, *Paraglenea* Bates and *Malloderma* Lacordaire. A new species, *Paraglenea dairanxingorum* Lin, You & Wang, sp. nov., is described from Hunan and Hubei Provinces, China. The new species is distinguished by its purplish-blue scales and median long, whitish, erect hairs on whole body. The type specimens were collected primarily using a sweeping net in wooded mountains in Fangziyacun, Madiyixiang, Yuanling County, Huaihua City, Hunan Province, as well as in Wudangshan, Shiyan City, Hubei Province. Although it resembles *Malloderma kuegleri* Holzschuh, 2010 in hair and scale morphology, it differs in male claw structure, elytral apex and puncture density. Additionally, *Paraglenea jianfenglingensis* Hua, 1985 is newly combined as *Malloderma jianfenglingense* (Hua, 1985) comb. nov., with a new distribution record from Guangxi, China. *Malloderma pascoei* Lacordaire, 1872, is recorded from Myanmar for the first time. Detailed comparisons between *P. dairanxingorum* Lin, You & Wang, sp. nov. and *Malloderma kuegleri* Holzschuh, 2010; *Malloderma jianfenglingense* (Hua, 1985) comb. nov. and *Malloderma pascoei* Lacordaire, 1872, are provided.

## Simple Summary

A remarkable new species of the genus *Paraglenea* Bates, *P. dairanxingorum* Lin, You & Wang, sp. nov., is described from Hunan and Hubei Provinces, China. This species is very beautiful due to its purple coloration, which is not very common for beetles. The purple color is formed by shining scales, sometimes giving a bluish appearance. Additionally, the beetle’s body is covered with soft white hairs, making it superficially similar to *Malloderma kuegleri* Holzschuh, 2010. This study also examines three species of the genus *Malloderma* Lacordaire, all characterized by long light-colored hairs and iridescent scales, and provides a detailed comparative analysis of their distinguishing features.

## 1. Introduction

The genus *Paraglenea* Bates, 1866, was described for *Glenea fortunei* Saunders, 1853, and the second species was *Paraglenea swinhoei* Bates, 1866 [1]. Gressitt (1951) added the third species, *P. atropurpurea*, from Fujian, China [2], and Breuning (1952) described four taxa: *P. chapaensis*, *P. latefasciata* and *P. swinhoei continentalis* from Vietnam and *P. transversefasciata* from Thailand [3]. Hua (1985) described the seventh species *P. jianfenglingensis* from Hainan Island of China [4], and Lin et al. (2017) transferred *Eutetrapha virides* Pu & Jin, 1991 [5], to this genus [6]. It currently includes 10 valid species and subspecies distributed across East and Southeast Asia, including China, Japan, Vietnam, Laos and Thailand [7]. In this paper, one remarkable new species, *P. dairanxingorum* Lin, You & Wang, sp. nov., is described from Hunan and Hubei Provinces of China.

The genus *Malloderma* Lacordaire, 1872 [8], currently includes three species distributed in China, Vietnam, Laos, India and Bhutan [7]. Fresh specimens of this genus are quite rare, so we only reported a small number of specimens in this study. *Paraglenea jianfenglingensis* Hua, 1985, is newly combined into the genus *Malloderma* Lacordaire, 1872, as *Malloderma jianfenglingense* (Hua, 1985) comb. nov. and newly reported from Guangxi, China. Meanwhile, the type species *Malloderma pascoei* Lacordaire, 1872, is recorded in Myanmar for the first time.

## 2. Materials and Methods

The male genitalia were prepared by removing the genitalia with forceps from fresh specimens without removing the abdomen, and clearing them in 10% KOH at room temperature for 16~24 h. Genitalia were photographed submerged in ethyl alcohol and subsequently preserved in polyethylene genitalia vials filled with glycerin and pinned under the specimens.

Habitus images were taken using a Canon EOS 7D camera with a Canon Macro 100 mm macro lens. Images of the same object at different focal planes were combined using Helicon Focus 8 stacking software. Adobe Photoshop CS6 was used for postprocessing. The terminalia were photographed with a Keyence VHX-1000C large-depth-of-field 3D digital microscope.

The specimens studied are deposited in the following institutional museums and private collections; abbreviations as shown in the text:

**CDC**—Collection of Chao Dai, Xupu, Huaihua, Hunan, China;

**CFF**—Collection of Fan Fu, Wuhan, Hubei, China;

**CWD**—Collection of Dong Wen, Qingdao, Shandong, China;

**CWLY**—Collection of Ling-Yun Wang, Taizhou, Jiangsu, China;

**CXKZ**—Collection of Ke-Zhen Xue, Changsha, Hunan, China;

**CYRJ**—Collection of Ren-Jie You, Xupu, Huaihua, Hunan, China;

**DHCO**—Daniel Heffern Collection, Houston, Texas, USA;

**IZCAS**—Institute of Zoology, Chinese Academy of Sciences [=NACRC National Animal Collection Resource Center], Beijing, China;

**MNHN**—Muséum national d’Histoire naturelle, Paris, France;

**MYNU**—Invertebrate collection of Mianyang Normal University, Mianyang, Sichuan, China;

**SYSU**—National Animal Collection Resource Center Sun Yat-sen University (The Museum of Biology), Guangzhou, China.

This article is registered in ZooBank under the following address:

urn:lsid:zoobank.org:pub:BA8C78BA-CF81-4F63-94EB-E778B0E744EC

## 3. Results

*Paraglenea dairanxingorum* Lin, You & Wang, sp. nov.

urn:lsid:zoobank.org:act:91646AFD-AFE8-4F3D-A629-B6FB382293C5

**Figure 1:**
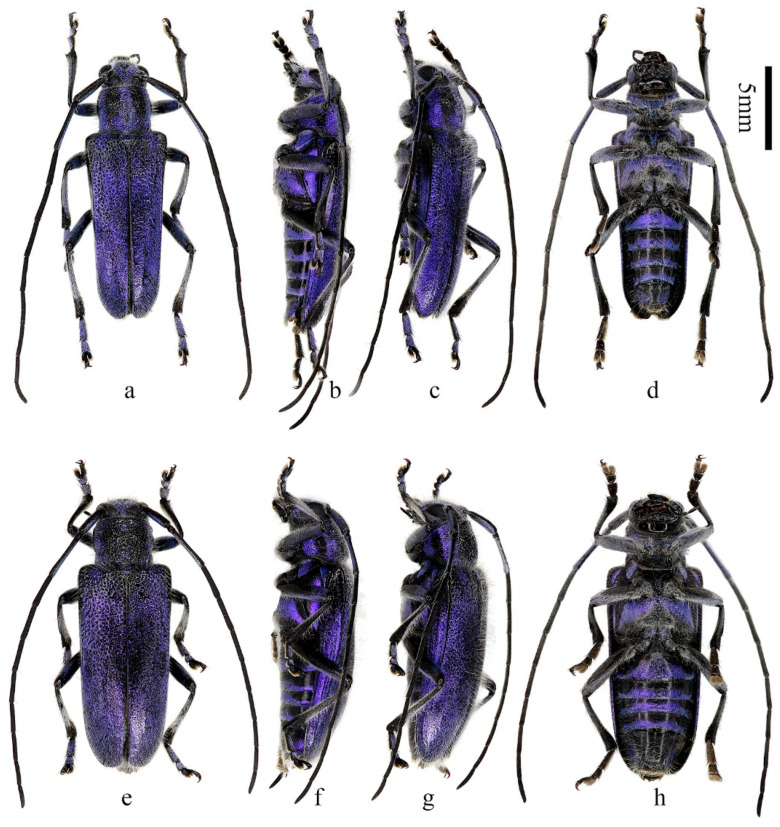
Habitus of *Paraglenea dairanxingorum* Lin, You & Wang, **sp. nov.** (**a**–**d**) Holotype, male, from Hunan. (**e**–**h**) Female, paratype, from Hunan. (**a**,**e**) Dorsal views. (**b**,**c**,**f**,**g**) Lateral views. (**d**,**h**) Ventral views. Scale bar: 5 mm.

**Figure 2:**
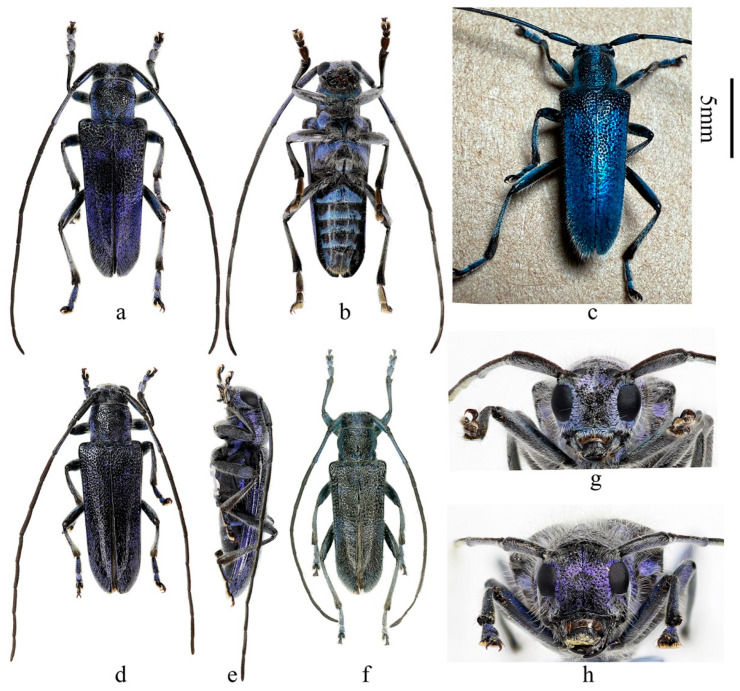
Habitus of *Paraglenea dairanxingorum* Lin, You & Wang, **sp. nov**., showing the variety of colors. (**a**–**g**) Male. (**h**) Female. (**a**–**e**,**g**,**h**) From Hunan. (**f**) From Hubei. (**a**,**c**,**d**,**f**) Dorsal views. (**e**) Lateral view. (**b**) Ventral view. (**g**,**h**) Head, frontal views. Scale bars for (**a**–**f**): 5 mm.

**Figure 3:**
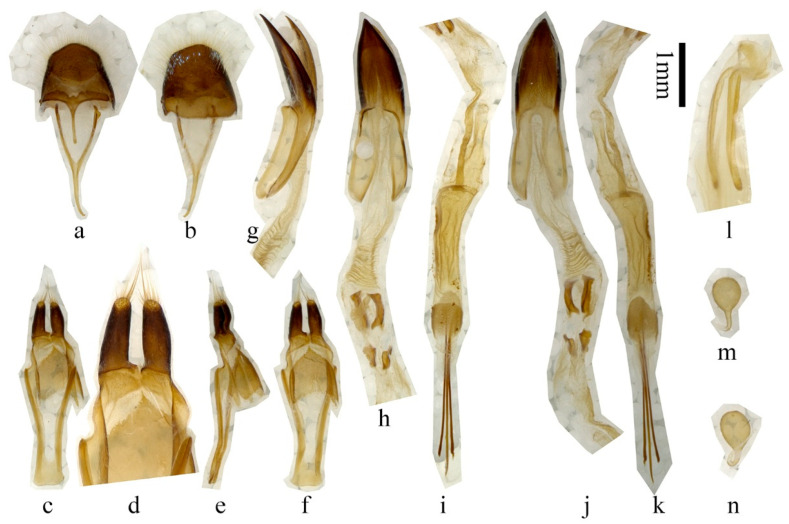
Terminalia of *Paraglenea dairanxingorum* Lin, You & Wang, **sp. nov**. (**a**–**k**) Male. (**l**–**n**) Female. (**a**,**b**) Tergite VIII with sternites VIII and IX. (**c**–**f**) Tegmen. (**g**–**k**) Median lobe and internal sac. (**l**) Ovipositor (**m**,**n**) Spermathecal capsule. (**a**,**c**,**d**,**h**,**i**) Ventral views. (**b**,**f**,**j**,**k**) Dorsal views. (**e**,**g**) Lateral views. Scale bars for (**a**–**c**,**e**–**k**): 1 mm.

**Figure 4:**
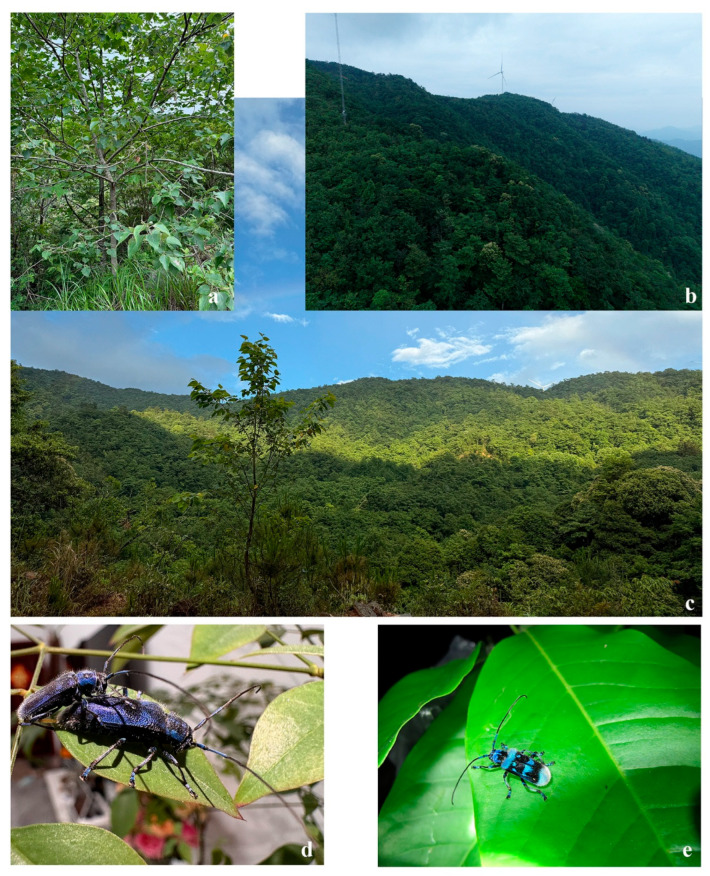
Habitats of *Paraglenea dairanxingorum* Lin, You & Wang, **sp. nov**., and *Malloderma jianfenglingense* (Hua, 1985) **comb. nov.** (**a**–**d**) *Paraglenea dairanxingorum.* (**a**) The tree that some type specimens were collected from. (**b**) The environment of type locality. (**c**) Type locality: Hunan, Huaihua City, Yuanling County, Madiyixiang, Fangziyacun, wooded mountain. (**d**) A mating pair, in the lab. (**e**) A living *Malloderma jianfenglingense* on a leaf, from Guangxi.

**Figure 5:**
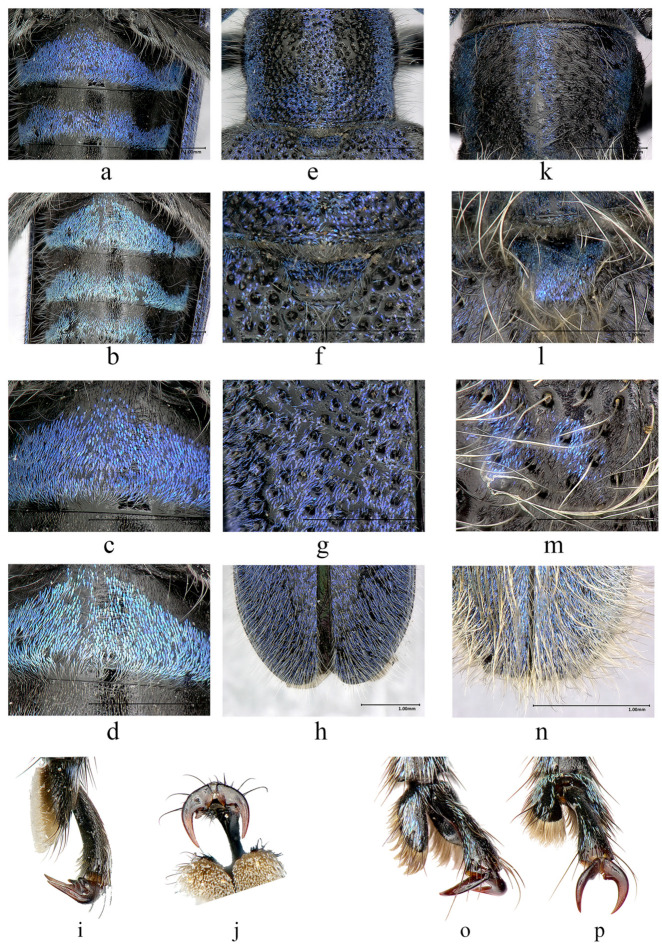
Comparative characteristics of *Paraglenea dairanxingorum* Lin, You & Wang, **sp. nov.**, and *Malloderma kuegleri* Holzschuh, 2010. (**a**–**j**) *Paraglenea dairanxingorum*. (**k**–**p**) *Malloderma kuegleri*. (**a**–**d**) Abdominal ventrites, showing the color variety. (**a**,**c**) Purplish individual. (**b**,**d**) Bluish individual. (**e**,**k**) Pronotum. (**f**,**l**) Scutellum. (**g**,**m**) Elytra, basal part with colorful scales. (**h**,**n**) Elytral apex, showing the long, erect hairs. (**i**,**o**) Male claws of mesotarsi. (**j**,**p**) Male claws of metatarsi. (**i**,**j**) Appendiculate claw. (**o**) Left claw of mesotarsi, appendiculate with small teeth on outer side. (**p**) Simple claw. Note: Not all pictures are exactly to scale.

Figure 1a–h, Figure 2a–h, Figure 3a–n, Figure 4a–d and Figure 5a–j.

Chinese common name: 戴氏然星双脊天牛.

**Description:** Body length: 10.0–18.6 mm, humeral width 3.0–6.0 mm. Body black, covered with dense erect white hairs and metallic purple (Figure 1a–h, Figure 2a,b and Figure 5a,c,e–h) to blue (Figure 2c and Figure 5b,d) scales except some black markings. The following black markings more or less stable: two longitudinal maculae on pronotal disc, and one smaller macula on each lateral side of prothorax; a larger sized transverse marking at base and a smaller and obscure one before middle of elytra, both without distinct shapes and borders (Figure 1a, Figure 2c and Figure 4d); basal part of ventrites II to V usually black (Figure 1d,h and Figure 2b). While the other black markings are caused by losing the metallic scales therefore unstable in shape and size. In some individuals, the pronotum mostly to totally black (Figure 1e and Figure 2a,d), so do the elytra (Figure 2d,f). Antennae black, sparsely pilose below, underside of basal three antennomeres covered with metallic purple scales. Whole scutellum covered with metallic purple scales. Side of elytra covered by metallic purple scales except carinae (Figure 1b,c,f,g and Figure 2e). Ventral surface densely clothed with metallic purple or blue scales, with few black spots. Legs black, covered with white hairs and some purple pubescence, tarsi with black hairs especially at sides.

Inferior eye lobe three times as long as gena in male (Figure 2g), or slightly longer than gena in female (Figure 2h). Antennae longer than body, male longer than female. Antennomere ratio: male: 10: 1: 12: 10: 10: 10: 9: 9: 8: 8: 10; female: 8: 1: 12: 10: 10: 9: 8: 8: 7: 6: 7. Elytron with punctures larger than that of pronotum (Figure 5e,f,g), both are very dense. Elytron with two lateral carinae (Figure 1b,c,f,g and Figure 2e), rounded apically (Figure 5h). Male claws appendiculate (Figure 5i,j). Females claws simple (Figure 5p).

**Male genitalia** (Figure 3a–k): Tergite VIII (Figure 3a,b) slightly broader than long, apex rounded, with dense and fine whitish setae, and some sparse metallic purple to blue scales (Figure 3b). Spiculum gastrale slightly subequal to ringed part of tegmen in length; spiculum relictum shorter than one half of spiculum gastrale. Tegmen (Figure 3c–f) about 2.9 mm in length; lateral lobes slender, each about 0.54 mm long and 0.17 mm wide (Figure 3d); apex with fine setae, some of which are longer than half of lateral lobes; median lobe slightly curved (Figure 3g), subequal to tegmen in length; median struts about half length of median lobe (Figure 3j); apex of ventral plate (Figure 3h) sharply pointed; endophallus more than triple length of median lobe, with two pairs of basal plate-like sclerites (Figure 3h,j, located far behind apex of median struts), two bands of indistinct supporting armature before the plate-like sclerites, and three rod-like sclerites at end (Figure 3i,k), two longer ones each about 2.48 mm, shorter than tegmen, short one about 2.01 mm. Female genitalia: ovipositor as Figure 3l, spermathecal capsule (Figure 3m,n) composed of an apical orb and a short but strongly curved stalk, strongly sclerotized part of stalk shorter than apical orb in length. Spiculum ventrale longer than abdomen. In our observation, spiculum ventrale measured 9.3 mm for one adult compared with abdomen, which measured 7.6 mm in ventral view.

**Diagnosis:** The new species is quite different from its congeners by the purplish or bluish scales and the median long whitish erect hairs on the body. It is definitely belonging to the genus *Paraglenea* Bates, 1866 by the three key characters: (1) male claws all appendiculate, female claws simple; (2) elytral with two lateral carinae; (3) elytral apex rounded [1,6]. The new species is similar to *Malloderma kuegleri* Holzschuh, 2010 by long and dense whitish erect hairs and purplish scales [9], but differs by the different male claws, which combined them into two genera. The new species with all male claws appendiculate (Figure 5i,j, the same kind to Figure 144 in [6]), which is the character of the genus *Paraglenea* Bates, 1866. While the genus *Malloderma* Lacordaire, 1872 have male claws with only anterior claws of pro- and mesotarsi appendiculate with small teeth, posterior claws of pro- and mesotarsi without teeth (Figure 5o, the same kind to Figure 146a–c in [6]), and claws of metatarsi simple (Figure 5p). Their comparative characteristics are summarized in Table 1.

**Type material. HOLOTYPE: CHINA**: ♂ (Figure 1a–d), Hunan, Huaihua City, Yuanling County, Madiyixiang, Fangziyacun, wooded mountain (湖南省怀化市沅陵县马底驿乡方子垭村山林, Figure 4a–c), alt. ca. 1100 m, 21.V.2025, leg. Chao Dai (IZCAS). **PARATYPES: CHINA:** 1 ♂1♀ (Figure 1e–h), same data to holotype; 2♂♂2♀♀, same data to holotype but 26.V.2025; 1♂1♀, same data to holotype but deposited in MYNU; 2♂♂1♀, same data to holotype but 17.VI.2025, deposited in MYNU; 2♂♂2♀♀, same data to holotype but deposited in CDC; 1♂1♀, same data to holotype but 26.V.2025 and deposited in CDC; 1♂1♀, same data to holotype but 26.V.2025 and deposited in CWD; 2 ♂♂, same data to holotype but 17.VI.2025 and deposited in CFF and CXKZ respectively; 4♂♂1♀, same data to holotype but 22.V.2025, leg. Ren-Jie You (CYRJ); 1♂, same data to holotype but 16.V.2025, leg. Ren-Jie You (CYRJ). 2♂♂ (Figure 2f), Hubei, Shiyan City, Wudangshan, Wuyaling (湖北十堰武当山乌鸦岭), alt. 900 m, 27.V.2024, leg. Ling-Yun Wang (CWLY).

**Distribution:** China: Hubei, Hunan.

**Etymology:** The new species is dedicated to three persons from the collector’s family, Mr. Chao Dai who collected most of the type specimens, and his two sons, An-Ran Dai and An-Xing Dai. “dai” is their family name, “ran” and “xing” represent An-Ran and An-Xing. Based on ICZN 11.9.1.3, “*dairanxingorum*” is a noum in the genitive case formed by adding -*orum* for the names of one man and two boys.

**Remarks:** Most of type specimens were caught by sweeping net when they were flying in the air, where the environment as shown on Figure 4b,c. Two specimens were collected from the tree *Populus* sp. on Figure 4a, and three specimens from *Tilia* sp., but they were occasionally rest on the leaves. Therefore the host plant is unknown to us. One mating pair were observed in lab condition (Figure 4d) on 27 May 2025, the male used fore and mid legs to hold the female, while the hind legs usually on plants or in the air. The specimens were collected from 21 May to 17 June, so May and June should be its adult active period.

 

***Malloderma pascoei Lacordaire*** **, 1872**

Figure 6g,h.

Chinese common name: 白毛天牛.

*Malloderma pascoei* Lacordaire, 1872: 842, note 1 [8]. Type locality: India: Indes orientales.

*Malloderma pascoei* m. *tonkinea* Pic, 1932: 151 [10]. Type locality: Vietnam: Tonkin: Chapa.

*Malloderma pascoei*: Aurivillius, 1923: 494 (catalogue) [11]; Löbl and Smetana 2010: 327 (catalogue) [12]; Lazarev, 2019: 171 (catalogue) [13]; Danilevsky 2020: 470 (catalogue) [14].

**Figure 6:**
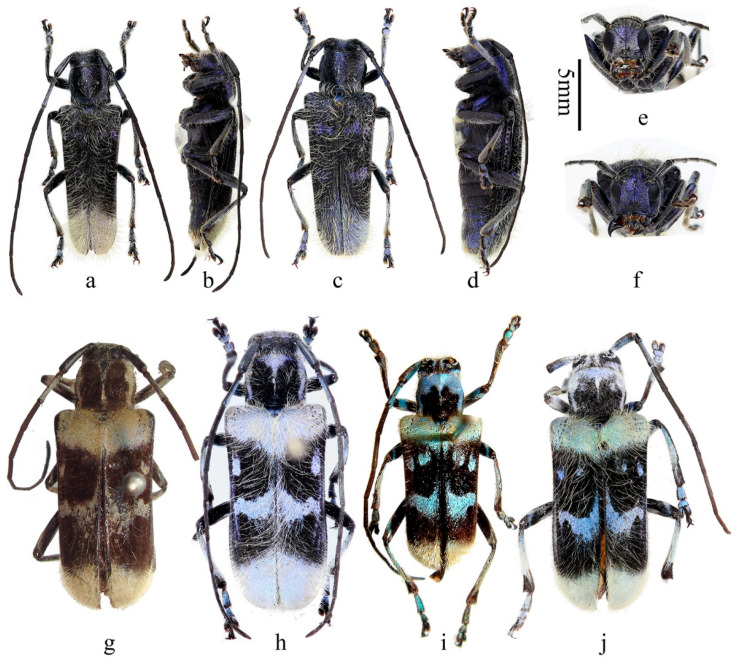
Habitus of *Malloderma* spp. (**a**–**f**) *Malloderma kuegleri* Holzschuh, 2010, paratypes, from Laos. (**g**,**h**) *Malloderma pascoei* Lacordaire, 1872. (**g**) Holotype, female, from India. (**h**) From Myanmar. (**i**,**j**) *Malloderma jianfenglingense* (Hua, 1985) **comb. nov.**, from China, Hainan. (**i**)Holotype. (**a**,**b**,**e**,**h**,**i**) Male. (**c**,**d**,**f**,**g**,**j**) Female. (**a**,**c**,**g**–**j**) Dorsal views. (**b**,**d**) Lateral views. (**e**,**f**) Head, frontal views. Scale bar: 5 mm.

**Examined material:** Holotype of *Malloderma pascoei* Lacordaire, ♂ (Figure 6g), Ind. Or. (Indes Orientales, means East India), (MNHN, ex. Musaeo Mniszech, ex. Coll. James Thomson, 1952); syntype of *Malloderma pascoei* m. *tonkinea* Pic, 1♀, Tonkin, Chapa, 22 June 1918, leg. Jeanvoine (MNHN, ex. Coll. M. Pic).

**Vietnam:** 1♀, Yen Bai Prov., Nghia Lo, April 2017, leg. local coll. (DHCO).

**India**: 2♂♂3♀♀, British Bootang, 1898, leg. L. Lurel (MNHN, ex. Coll. R. Oberthur, 1952); 1♀, British Bootang, 1898, leg. L. Lurel (MNHN, ex. Coll. M. Pic); 1♀, British Bootang, 1898, leg. L. Lurel (MNHN, ex. Coll. R. P. Belon & Coll. A. Argod, 1931); 2♂♂1♀, British Bootang, 1899, leg. L. Lurel (MNHN, ex. Coll. R. Oberthur, 1952); 1♂1♀, British Bootang, 1898, leg. L. Lurel (MNHN); 2♀♀, British Bootang, 1899, leg. L. Lurel (MNHN); 1♂9♀♀, British Bootang, Padong, 1914, leg. L. Lurel (MNHN, ex. Coll. R. Oberthur, 1952); 2♂♂7♀♀, British Bootang, Padong, 1914, leg. L. Lurel (MNHN); 1♂1♀, Pedong, leg. A. Desgodins (MNHN, ex. Coll. M. Pic); 2♂♂4♀♀, Pedong, leg. A. Desgodins (MNHN); 1♀, Pedong, leg. A. Desgodins (MNHN, ex. Coll. R. P. Belon & Coll. A. Argod, 1931); 1♀, Pedong, leg. A. Desgodins (MNHN, ex. Coll. Léon Fairmaire, 1906); 1♂, North India, (MNHN, ex. Coll. Musaeo James Thomson, 1952); 1♀, Bootan Indep.,1913, leg. Native collector (MNHN); 1♀, Inde Anglaise Pedong Région de Darjecling, Chasseurs indigenes, 1935 (MNHN, ex. Coll. R. Oberthur, 1952); 1♀, Inde Anglaise Pedong Région de Darjecling, Chasseurs indigenes, 1931 (MNHN, ex. Coll. M. Pic); 1♀, N. W. India (MNHN); 1♂, Sikkim Kurseong, leg. P. Bretaudeau (MNHN, ex. Coll. R. Oberthur, 1952); 1♂, Sikkim Aband (MNHN, ex. Coll. R. Oberthur, 1952).

**Myanmar**: 2♀♀ (Figure 6h), Shan Highland Mong Hkok, 1–22 May 2005 (DHCO).

**Remarks:** This is the type species of the genus *Malloderma*. It was recorded from India [8], Vietnam [10], and Bhutan [12,13]. We had examined material from Vietnam, India and Myanmar. It is recorded from Myanmar for the first time. While the record from Bhutan by Löbl and Smetana (2010) [12] and Lazarev (2019) [13] were very doubtful. They did not mention any material [12,13], it was very possible based on misunderstanding of the “British Bootang”. Some authors treated it as a place from Bhutan [12], however, it is infact Darjeeling District. Therefore we did not include Bhutan in the distribution list.

 

***Malloderma jianfenglingense* (Hua**, **1985) comb. nov.**

Figure 4e and Figure 6i,j.

Chinese common name: 尖峰白毛天牛.

*Paraglenea jiangfenglingensis* [sic] Hua, 1985: 87, Figures 1 and 2 [4]. Type locality: China: Hainan.

*Paraglenea jianfenglingensis*: Hua et al., 1993: 163, 295, pl. XXII, 360a. (justified emendation) [15]; Hua, 2002: 222 (catalogue) [16]; Hua et al., 2009: 240, 384, 440, Figure 1187 [17]; Löbl and Smetana 2010: 329 (catalogue) [12]; Lin and Tavakilian 2019: 397 (catalogue) [18]; Danilevsky 2020: 472 (catalogue) [14].

**Examined material:** Holotype (Figure 6i), ♂, Hainan, Jianfengling (海南尖峰岭), 27 April 1982, leg. Mao-Bin Gu by hand (SYSU, Ce-007151); 1♀ (Figure 6j), Hainan, Jianfenglingding (海南尖峰岭顶), 14 April 1983, leg. Mao-Bin Gu by hand (IZCAS). 1 individual (Figure 4e), Guangxi (a living beetle picture from Zhi-Lin Chen in 12 May 2011).

**Distribution:** China: Hainan, Guangxi (new record).

**Remarks:** This is a rare species, with only one holotype, one allotype in the original literature, and no more specimens reported after that. In this paper, we reported another female specimen deposited in IZCAS (Figure 6j), and a living picture from Guangxi (Figure 4e, new distribution record). Based on the limited material, we have enough reasons to proposed the new combination *Malloderma jianfenglingense* (Hua, 1985) based on three key characters: (1) male claws are *Malloderma* style (with only anterior claws of pro- and mesotarsi appendiculate with small teeth, posterior claws of pro- and mesotarsi without teeth, Figure 5o), not *Paraglenea* style (all appendiculate, Figure 5i,j); (2) body covered with very long light colored hairs, just the same to the type species *Malloderma pascoei* Lacordaire, 1872; (3) the body shape, ratios of pronotum/elytra, elytral width/length etc, are the same to the type species. While we do not have enough reasons to synonymized it with or downgraded it to be a subspecies of *Malloderma pascoei* Lacordaire, 1872. They are quite similar to each other but can be easily separated by: (1) the black maculae on pronotum, larger and nearly reaching apical margin for *M. pascoei* (Figure 6g,h), smaller and quite far from apical margin, only partly extending to middle for *M. jianfenglingense* (Figure 6i,j); (2) the light color maculae in the first black bank on basal half elytra, only one near the lateral margin for *M. pascoei* (Figure 6g,h), while with two maculae for *M. jianfenglingense* (Figure 6i,j), the other one located near suture and a little anterior than the similar one for *M. pascoei*.

## 4. Discussion

About the specific names “*jianfenglingensis*” vs. “*jiangfenglingensis*”:

Though Hua (1985) misspelled “*jiangfenglingensis*” in the original paper [4] and on the type label, it should be corrected as “*jianfenglingensis*”. There is not a locality named “Jiangfengling” in Hainan Island, and it is definitely a misspelling of “Jianfengling”. On page 88, Chinese “尖峰岭” (Jianfengling) was repeated twice for type specimen information, and “尖峰双脊天牛” (which means “jianfeng *Paraglenea*”) was applied twice for figure legends of holotype and allotype, and the English abstract contained two instances of “Jianfengling of Hainan Island”. People from South China sometimes mix words pronounced with or without a “g”. Hua et al., 1993, made the justified emendation by correctly repeating “*jianfenglingensis*” once on page 50, twice on page 163, twice on page 295, and once again on page 318 [15]. “*jianfenglingensis*” was used once in Hua (2002) [16], six times in Hua et al. (2009) [17], three times in Lin and Tavakilian (2019) [18] and twice in the two versions of the Catalogue of Palaearctic Coleoptera by Löbl and Smetana (2010) [12] and Danilevsky (2020) [14].

The species name “*jianfenglingensis*”, after the type locality Mt. Jianfengling, was applied to five genera, four times by Li-Zhong Hua: *Paraglenea jianfenglingensis* Hua, 1985 [4]; *Yimnashaniana jianfenglingensis* Hua, 1986 [19]; *Procleomenes jianfenglingensis* Hua, 1986 [20]; *Anoplophora jianfenglingensis* Hua, 1989 [21]. Another instance is *Rhaphuma jianfenglingensis* Viktora & Liu, 2018 [22], while *Acrocyrtidus jianfeng* Viktora & Liu, 2018, was also named after the type locality Mt. Jiangfengling of Hainan Island [23].

The Titan database still uses “*jiangfenglingensis*” for *Paraglenea jianfenglingensis* [7]. It also uses the incorrect spelling “*Anoplophora jiangfenglingensis* Hua, 1989” [7], while all of the printed literature correctly uses “*Anoplophora jianfenglingensis* Hua, 1989” [12,14,15,16,17,18,21].

Li-Zhong Hua also used “*jianfenglingensis*” to name another two insects, *Kishinouyeum jianfenglingense* Hua, 1984 (original spelling was *jianfenglingensis*, Mantodea) [24], and *Merodontina jianfenglingensis* Hua, 1987 (Diptera) [25].

## Figures and Tables

**Table 1:** Comparative characteristics of *Paraglenea dairanxingorum* Lin, You & Wang, sp. nov., and *Malloderma kuegleri* Holzschuh, 2010.

Characters	*Paraglenea dairanxingorum* Lin, You & Wang, sp. nov.	*Malloderma kuegleri* Holzschuh, 2010
Whitish, erect hairs	Soft and thin, median long (Figure 5e–h)	Thick, very long (Figure 5k–n)
Punctures	Denser (Figure 5e–g)	Sparser (Figure 5k–m)
Elytral apex	Rounded (Figure 5h)	Truncated (Figure 5n)
Color pattern	Almost entirely covered by purplish to bluish scales (Figure 1a–h and Figure 2c,g,h), without stable maculae on elytra (some black maculae were caused by loss of scales, such as Figure 2a,c)	Mostly black, only some parts covered by purplish scales, apical quarter of elytra apparently separated from basal parts, with light color, and basal dark color part of elytra with irregular purple maculae (Figure 6a–f)
Male claws of pro- and mesotarsi	Appendiculate claws (Figure 5i)	Anterior claws appendiculate with small teeth on outer side (Figure 5o)
Male claws of metatarsi	Appendiculate claws (Figure 5j)	Simple claws (Figure 5p)

## Data Availability

The original contributions presented in this study are included in the article. Further inquiries can be directed to the corresponding author.
